# Comparison of endoscopic band ligation devices used for colonic diverticular bleeding: in vivo animal study

**DOI:** 10.1002/jgh3.12445

**Published:** 2020-10-28

**Authors:** Yasutoshi Shiratori, Takashi Ikeya, Koyu Suzuki, Kazuki Yamamoto, Takaaki Yoshimoto, Ayaka Takasu, Noriaki Oguri, Takeshi Okamoto, Syuhei Okuyama, Koichi Takagi, Katsuyuki Fukuda

**Affiliations:** ^1^ Division of Gastroenterology St. Luke's International Hospital Tokyo Japan; ^2^ Department of Pathology St. Luke's International Hospital Tokyo Japan

**Keywords:** animal study, colonoscopy, endoscopy, endoscopic band ligation, gastrointestinal bleeding, pathology, therapeutic endoscopy

## Abstract

**Background and Aim:**

Endoscopic band ligation (EBL), used for the treatment of colonic diverticular bleeding, has a lower rebleeding rate than endoscopic clipping. However, different devices are used in Japan and the Western countries; no animal studies have been conducted to elucidate the safety of such devices. We compared two EBL devices, the first used in Japan and the second used in Western countries.

**Methods and Results:**

The Japanese and Western EBL devices were compared by assessing the EBL safety at 40 sites in an animal model with a normal colon that is anatomically similar to the human colon. Macroscopic and pathological examinations were performed to evaluate the layer ligated by the band and the presence of perforation. The findings on day 1 and day 7 after EBL were compared. The ligated layer was the muscularis propria at 39 sites; the layer was not evaluated at one site where the band was unintentionally removed during the endoscopic procedure. Pathologically, there was no perforation at any of the assessed sites. There was no statistical difference in any of the pathological variables between the two devices or between days 1 and 7 after EBL. The total procedure time was significantly shorter with the Western EBL device.

**Conclusions:**

In this animal study, both evaluated devices were safe for EBL, without differences in the macroscopic and pathological variables after EBL. Ligation of the muscularis propria layer did not result in perforation.

## Graphical abstract

We performed endoscopic band ligation (EBL) on colons of pigs using two different EBL devices and compared the macroscopic and pathological findings to evaluate the efficacy and safety of the two EBL devices, the first used in Japan and the second used in Western countries. Our results indicated that both evaluated devices were safe for EBL. A suitable device can be selected according to the patients' clinical condition.

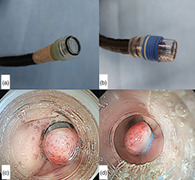

## Introduction

Colonic diverticular bleeding (CDB) is the most frequent cause of lower gastrointestinal bleeding. With the aging of the population, the incidence of CDB is expected to increase in the future.^1^, ^2^, ^3^ In Japan, a guideline for CDB treatment was developed in 2018.^4^ Several hemostatic methods for CDB treatment have been reported, including endoscopic band ligation (EBL), endoscopic clipping, end‐loop method, epinephrine injection, and heat coagulation, and they have been compared regarding successful hemostasis and early rebleeding rates.^5^, ^6^, ^7^ EBL is performed in two steps: First, the bleeding source is identified with the scope and marked with a clip; second, the EBL device is introduced, which is used to invert the diverticulum using a suction procedure and ligate it with the band.

Although EBL is more effective than endoscopic clipping in terms of the early rebleeding rate,^5^, ^6^, ^7^ mucosal ischemia caused by banding has been reported to cause EBL‐related adverse events, such as perforation or diverticulitis. The use of EBL for the treatment of CDB is increasing; however, reported postprocedural pathology related to the currently used EBL devices is limited to perforation cases.^8^, ^9^, ^10^ In the two reported cases, sigmoid colon perforation occurred 4 and 5 days post‐EBL despite an uneventful EBL procedure.^8^, ^9^ On the contrary, in a prior colonic surgery case, banding of the muscularis propria layer did not lead to perforation at 1 day after EBL.^5^ As mentioned above, the post‐EBL pathology has not been evaluated sufficiently, and the safety of the currently used devices needs to be confirmed.

One of the reasons for the widespread use of EBL in Japan is that Akimaru *et al*. conducted animal experiments on pigs to demonstrate the safety of the device.^11^ However, in that study, Ligation system (Ligation apparatus, HX‐21L‐1; Quick Loop, MAJ‐339; Olympus, Tokyo, Japan) was used, a device that is not currently widespread. Currently widely used in Japan is the EBL device (MD48912B EBL Device; Sumitomo Bakelite Co Ltd., Tokyo, Japan) that was approved by the Pharmaceutical Affairs for CDB and internal hemorrhoids released in August 2018. Witte *et al*. evaluated the Speedband Superview Super7 (Boston Scientific Co, Ltd., Natick, MA, USA), a device used to perform EBL in Western countries.^12^ Nonetheless, there are limited studies on EBL for CDB treatment in Western countries.

Considering that different EBL devices are used in Japan and Western countries and that neither of the currently used devices has been evaluated in animal studies, we aimed to investigate the safety, endoscopic views, and pathological changes after EBL between the two devices by conducting an in vivo animal study.

## Methods

### *Ethical considerations*

The study was approved by our Institutional Review Board (IVT 10‐06) and Animal Use Committee.

### *Study design and animals*

The Japanese EBL device (J‐EBL; MD48912B) and the Western EBL device (W‐EBL; Speedband Superview Super7) were used to perform in vivo animal experiments in two pigs weighing approximately 40 kg. The pig model was used because of the anatomical similarities with the human colon. The experiments were conducted at IV TeC Intervention Technical Center Co, Ltd., Kobe, Japan. The two devices' features are shown in Table 1.

**Table 1:** Features of the endoscopic band ligation devices

	J‐EBL	W‐EBL
Device	EBL Device (MD48912B)	Speedband Superview Super 7
Company	Sumitomo Bakelite Co., Ltd.	Boston Scientific Co., Ltd.
Height of ligator	5.0 mm	6.2 mm
Diameter of inner hood	11.8 mm	8.6 mm
Number of O‐rings that can be attached	1	7
Feature	Wide endoscopic view	Continuous banding is possible (equipped with 7 bands)
Available colonoscopy resource	OLYMPUS PCFQ260, PCF‐H290, PCF‐H290Z, CF‐H290, CF‐HQ290	OLYMPUS PCF‐Q260, PCF‐H290T, PCF‐H290, PCF‐H290Z, CF‐H290

EBL, endoscopic band ligation.

EBL was performed in two pigs under deep sedation and mechanical ventilation support. After the procedure, sedation was stopped, and the animals were observed for clinical signs.

**Figure 1:**
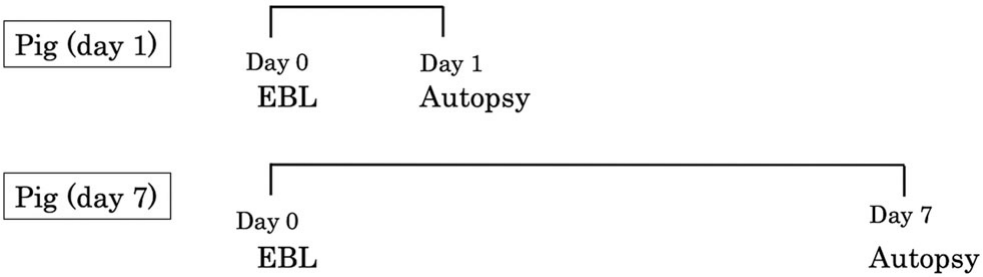
Time course for the day 1 and day 7 groups. In the day 1 group, autopsy was performed 1 day after endoscopic band ligation. In contrast, autopsy was performed 7 days after endoscopic band ligation in the day 7 group.

For temporal comparison, autopsy in the two pigs was performed at days 1 and 7 post‐EBL (Fig. 1). The band‐ligated sites were assessed macroscopically and pathologically. The macroscopic and pathological differences were evaluated between the devices or between day 1 and day 7 after EBL. In addition, the procedure time was compared between J‐EBL and W‐EBL.

### *EBL* *procedure*

**Figure 2:**
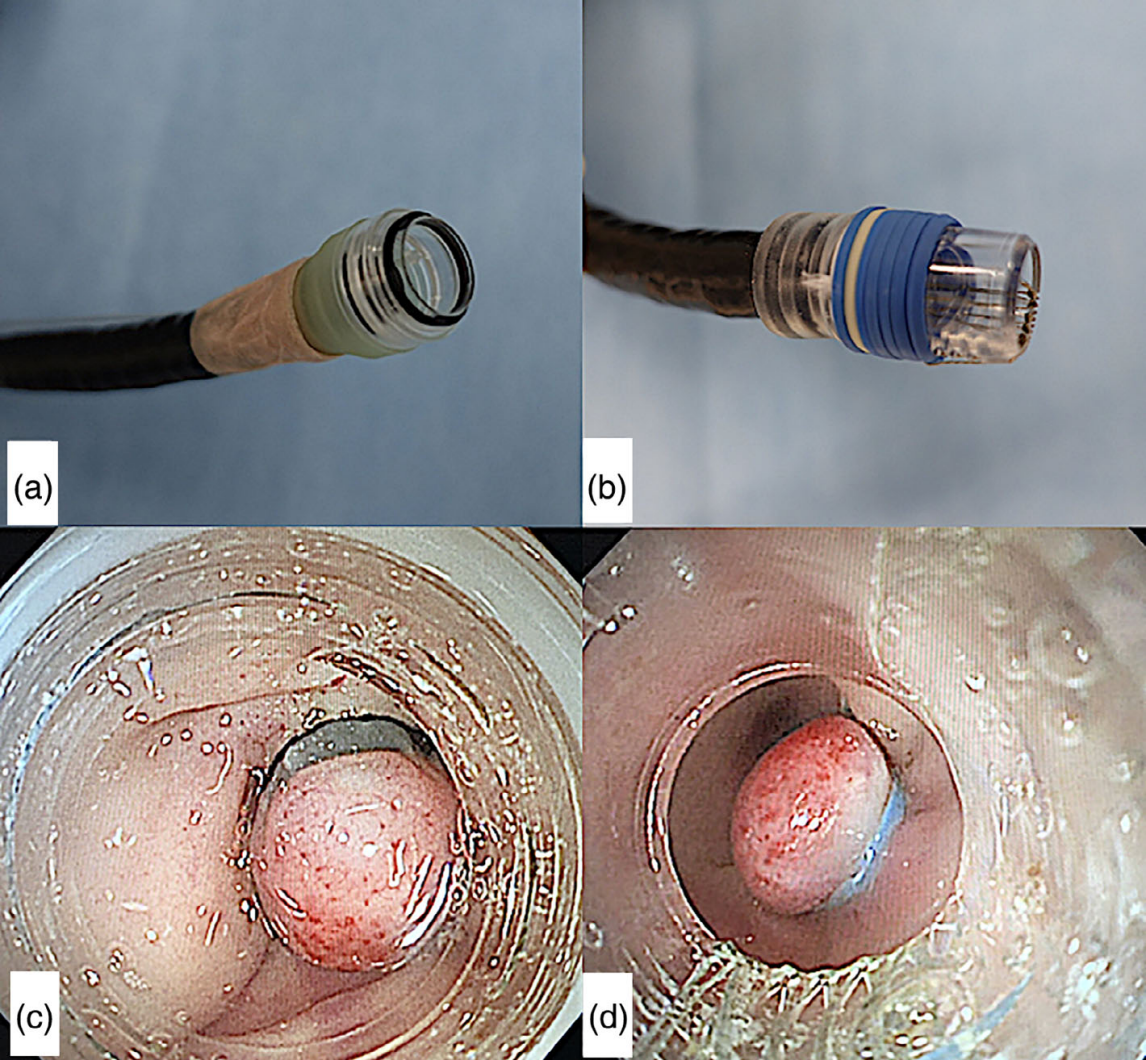
Images of the endoscopic band ligation devices. (a) Japanese endoscopic band ligation device; (b) Western endoscopic band ligation device; (c) endoscopic view of the Japanese endoscopic band ligation device; (d) endoscopic view of the Western endoscopic band ligation device.

EBL was performed by six endoscopists (two experts and four nonexperts) experienced in EBL. We defined an expert endoscopist as one who had conducted more than 1000 colonoscopies with board certification from the Japanese Gastroenterological Endoscopy Society; other endoscopists were defined as nonexperts. Before the procedure, an intestinal cleansing agent (polyethylene glycol, 2 L) was administered orally over 2 h. After sedative anesthesia was applied, rectal irrigation with clear water was additionally performed. The pigs were placed in the left lateral decubitus position, and colonoscopy was performed. The scope (GIF‐Q260J; Olympus Optical Co, Ltd., Tokyo, Japan) with an attached EBL device was inserted through the anus to 100 cm from the anal verge. The pigs' colon was too long to perform total colonoscopy, and there were no left‐ and right‐side sections anatomically. EBL was performed with the ligation sites 5 cm apart. Suction of the colonic mucosa into the ligator cup for approximately 2–3 s resulted in band release. When EBL was performed consecutively using the J‐EBL device (Fig. 2a), the scope was removed before each ligation to attach the next band. This was not required when the W‐EBL (Fig. 2b) device was used for sequential EBL because it had seven bands. We evaluated the differences in the endoscopic visual fields between the two devices (Fig. 2c,d).

### *Pathological analysis*

The EBL sites were evaluated by endoscopy before the autopsy was performed to confirm that the bands were in place. At autopsy, macroscopic observation was performed for the presence of surrounding inflammation and perforation. Subsequently, excision, fixation, and hematoxylin and eosin staining were performed by the pathologist. Pathological evaluation was carried out by our hospital pathologist. EBL‐involved layers, perforation, necrotic tissue at the ligation site, vascular ischemia, granulation, and neutrophil and monocyte infiltration were evaluated as the outcomes.

The thickness of the colonic wall in pigs is 3–5 mm and is not different from that in humans. The thickness of each layer (mucosa: 0.3 mm, submucosal layer: 0.6 mm, muscularis propria: 0.6 mm, subserosa: 1.8 mm, serosa: 0.2 mm) is also similar to that in humans.

### *Statistical analysis*

The EBL sites were categorized according to the device used and the time of evaluation (J‐EBL or W‐EBL and day 1 or day 7, respectively), and the findings were compared. Fisher's exact test was used for categorical and Mann–Whitney's test for continuous variables. Data management and statistical analyses were performed using Stata version 16 (Stata Co., College Station, TX, USA). *P*‐values less than 0.05 were considered statistically significant.

## Results

A total of 40 EBL procedures (20 with the J‐EBL and 20 with the W‐EBL; 20 by experts and 20 by nonexperts) were performed. No differences were observed based on the endoscopists' experience. The outcome is summarized in Table 2.

**Table 2:** Summary of the study results

	J‐EBL			W‐EBL			*P* value		
	Total	Day 1	Day 7	Total	Day 1	Day 7	Total	Day 1	Day 7
Number of EBL sites	20	10	10	20	10	10			
Total procedure time, min	98	50	48	64	34	30	<0.01	<0.01	<0.01
Insertion time to the deepest colonic portion, min	14	7	7	16	8	8	0.31	0.31	0.31
Remaining band, *n* (%)	19 (95)	9 (90)	10 (100)	20 (100)	10 (100)	10 (100)	0.31	0.30	1
Perforation, *n* (%)	0 (0)	0 (0)	0 (0)	0 (0)	0 (0)	0 (0)	NA	NA	NA
Adhesion to nearby intestine, *n* (%)	2 (10)	1 (10)	1 (10)	3 (15)	1 (10)	2 (20)	0.63	1	0.26
Ligated layer, MP/serosa/unknown	19/0/1	9/0/1	10/0/0	20/0/0	10/0/0	10/0/0	0.31	0.30	1
Necrosis at the ligature, *n* (%)	20 (100)	10 (100)	10 (100)	20 (100)	10 (100)	10 (100)	NA	NA	NA
Disruption of the blood vessel at the ligature, *n* (%)	14 (70)	7 (70)	7 (70)	15 (75)	7 (70)	8 (80)	0.72	1	0.63
Granulation at the ligature, *n* (%)	10 (50)	0 (0)	10 (100)	10 (50)	0 (0)	10 (100)	NA	NA	NA
Neutrophil infiltration, *n* (%)	10 (50)	0 (0)	10 (100)	10 (50)	0 (0)	10 (100)	NA	NA	NA
Monocyte infiltration, *n* (%)	10 (50)	0 (0)	10 (100)	10 (50)	0 (0)	10 (100)	NA	NA	NA

EBL, endoscopic band ligation; MP, muscularis propria; NA, not applicable.

### *Macroscopic evaluation*

**Figure 3:**
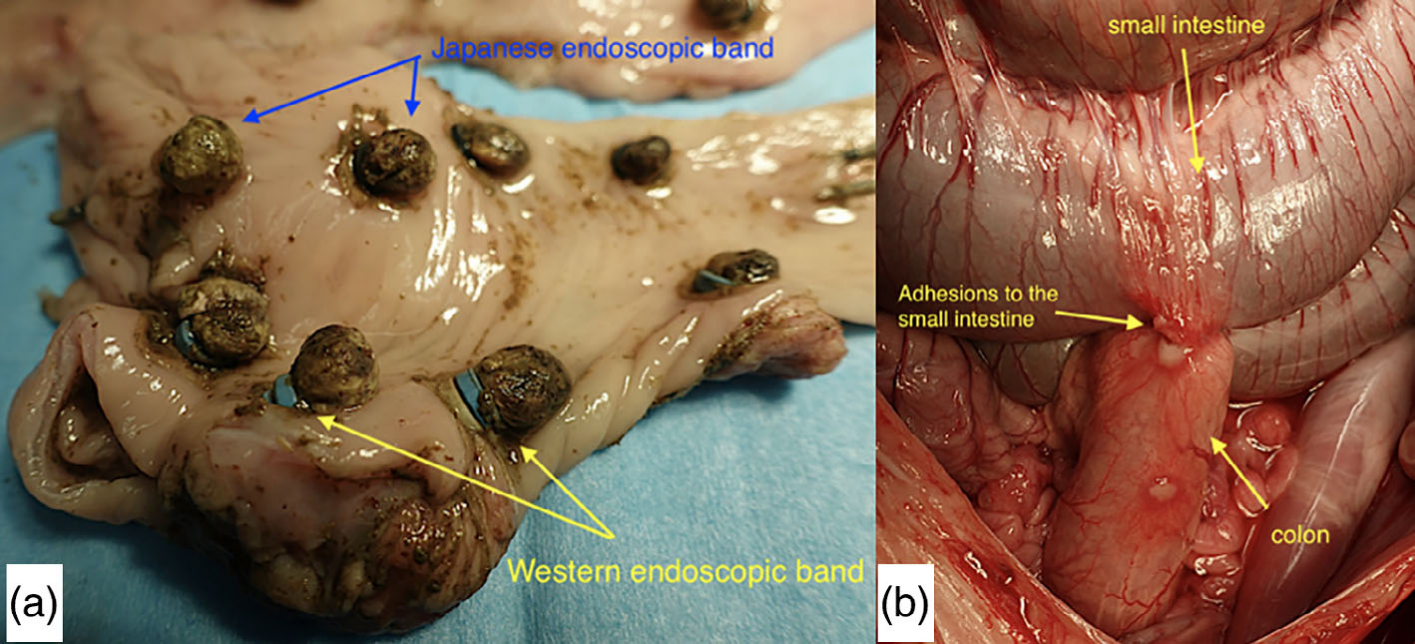
Macroscopic evaluation images. (a) Macroscopic evaluation of the resected colon; (b) macroscopic evaluation revealed adhesions with the adjacent small intestine.

At the time of macroscopic evaluation, the bands were in place in 39 of 40 (95%) sites (Fig. 3a). The band was dislodged at only one site (J‐EBL, day 1), at which the ligated tissue was unintentionally removed during the endoscopic procedure. Macroscopically evident perforation was not found at any of the EBL sites. At the time of autopsy, adhesions with the adjacent bowel were observed in two cases by J‐EBL and in three cases by W‐EBL (*P* = 0.63; Fig. 3b). No bowel narrowing or dilation was observed at the same site.

### *Pathological evaluation*

**Figure 4:**
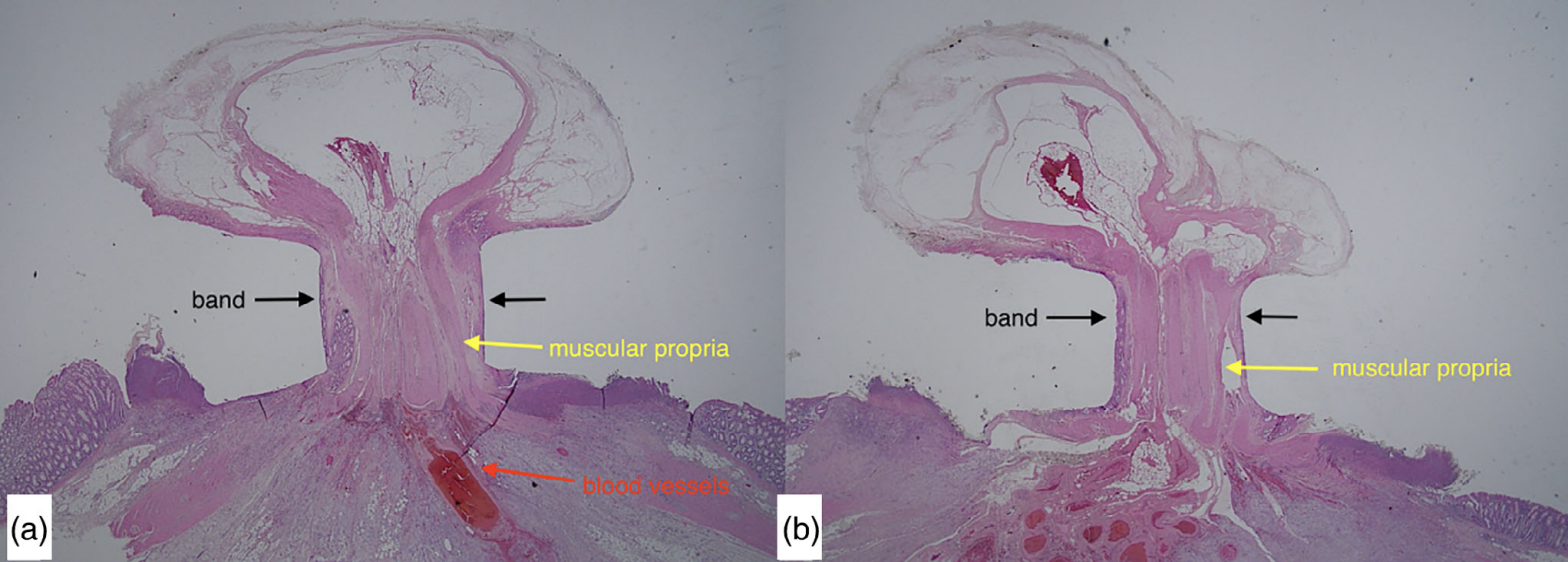
Microscopic image of a ligated section stained with hematoxylin and eosin (10×). A representative example of muscularis propria involvement. (a) Japanese endoscopic band ligation device; (b) Western endoscopic band ligation device.

**Figure 5:**
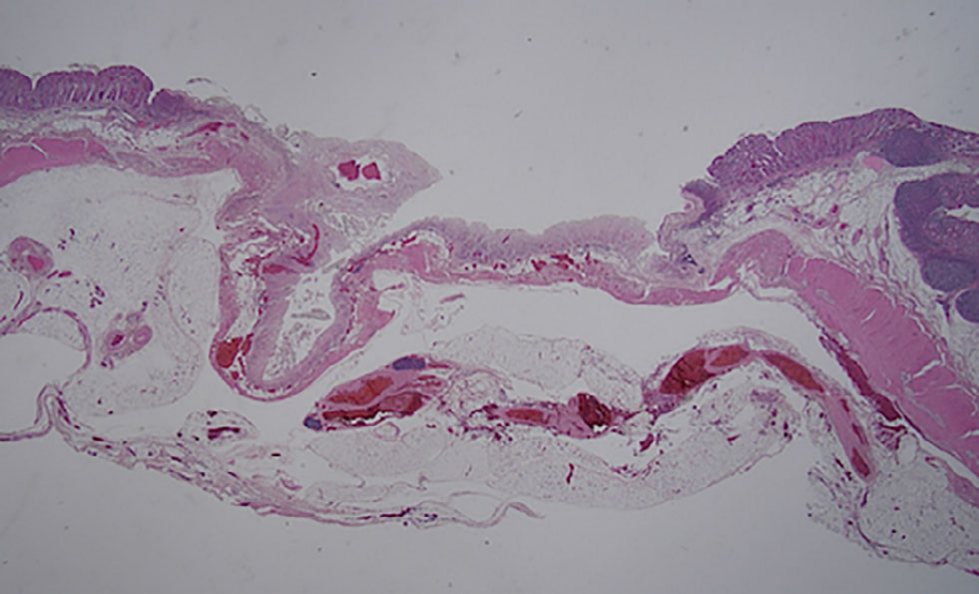
Microscopic image of the site where the band was dislodged. The muscularis propria layer was detached and a part of the subserosal layer was exposed.

Regarding the ligated layers, 39 sites that could be evaluated were ligated at the muscularis propria layer (Fig. 4a,b). The site where the band was dislodged could not be assessed for the ligated layer. However, at this site, the muscularis propria layer was detached, and the subserosal layer was exposed (Fig. 5). Necrosis at the ligation site was confirmed in all cases. Vascular disruption was found in 14 of 20 (70%) sites ligated by the J‐EBL and in 15 of 20 (75%) sites ligated by the W‐EBL (*P* = 0.72). There were no statistical differences between the two devices in terms of the presence of granulation tissue and neutrophil and monocyte infiltration; however, such histological findings were observed only on day 7 post‐EBL. Adhesions to the adjacent bowel and peritoneum were found in 2 of 20 (10%) and 3 of 20 (15%) ligated sites by the J‐EBL and the W‐EBL, respectively (*P* = 0.63).

### *EBL* *procedure*

All EBL procedures were performed completely. There were no sites at which EBL could not be performed. The total procedural time was significantly shorter with the W‐EBL than with the J‐EBL (68 min *vs* 98 min, *P* < 0.01). Although endoscopic visualization was better with the J‐EBL than with the W‐EBL (inner hood diameter: 11.8 mm *vs* 8.6 mm; Table 1, Fig. 2), the time taken for insertion in the deepest portion of the colon was not significantly different between the two devices (8 min *vs* 7 min, *P* = 0.31).

## Discussion

In this study, we performed EBL on normal colons of pigs using two different EBL devices and compared the macroscopic and pathological findings. There were no significant differences in any of the evaluated outcomes.

Until now, there have been only a few studies on EBL with pathological examination.^5^, ^8^, ^13^ As summarized in Table 3, the previous studies were performed ex vivo, and the devices used are currently not widespread. In addition, the follow‐up period in one of the studies (number 3 in Table 3) was only 1 day. The present study is the first in vivo animal‐based study using EBL devices that are widely used in Japan and Western countries. We used EBL devices in human‐like pig colons and not in human diverticula, and intestinal peristalsis in pigs was suppressed under general anesthesia, which differs from actual clinical practices. We therefore consider these study limitations. However, as stated in a prior report,^5^ because the normal colonic mucosa around the diverticulum is compressed by the band, evaluation of the banded layer is important for assessing the safety of the EBL device, regardless of the diverticulum.

**Table 3:** Previously reported studies on endoscopic band ligation devices

No	Author	Target	Device	Outcome
1	Barker *et al*.	Human, fresh resected colon	Wilson cook medical six shooter (Cook Medical, Co, Bloomington, USA)	The right colon was ligated at the muscularis propria layer, while the left colon at the submucosal layer.
2	Akimaru *et al*.	Pig	Ligation system (Olympus Co, Tokyo, Japan)	The muscularis propria layer was ligated. No perforation 14 days after treatment.
3	Ishii *et al*.	Human, postoperative ascending colon	EVL device (Sumitomo Bakelite, Tokyo, Japan)	The muscularis propria layer was ligated. There was no perforation.

EVL, endoscopic variceal ligation.

Regarding the evaluated outcomes and the macroscopic and pathological differences, the colons of the pigs were 3–5 mm thick and similar in the proportion of each layer to the human colon. With both devices, the muscularis propria layer was involved, but there was no perforation or serosal ligation observed in this study. Ligation of the muscular layer did not seem to lead to perforation. This result supports the previously reported hypothesis that muscularis propria layer ligation does not cause perforation. However, during the endoscopy, the endoscopic hood made contact with one of the banded sites, causing the ligated tissue to detach (W‐EBL, day 1). At the same site, when autopsy was performed to confirm that the bands were in place, the muscularis propria layer had detached, and the subserosal tissue was exposed. This indicates that early removal of the ligated portion causes shedding of the banded mascularis propria and involves the risk of perforation. Hence, the banded sites should not be removed. There were also several cases of adhesions with the adjacent small intestine, but the pigs had no clinical manifestations. Although the adhesions were accompanied by inflammatory fibrosis between the EBL site and the peritoneum of the adjacent bowel, the bowel was not pulled into the band. The adhesions did not affect the small bowel lumen; there was no bowel narrowing or caliber change. Although the pig colon is anatomically similar to the human colon, in our study, it was spiral‐shaped and 4–4.5 m long, and the small intestine was 16 m long and closely adjacent to the abdominal cavity. We believe that the pig's large intestine allowed for easy aspiration of the surrounding tissue during EBL.

In the comparison between day 1 and day 7 post‐EBL, there were no cases of spontaneous band dropout in both groups; the bands remained in place until day 7. The presence of necrosis and vascular disruption were not statistically different between the groups. However, granulation and infiltration of neutrophils and monocytes were only observed on day 7. It has been reported that granulation usually occurs after day 3.^14^ The granulation tissue that replaces the band was not sufficiently developed on day 7, suggesting that more time is needed for granulation to completely cover the ligation site and create a scar. Based on these findings, endoscopic removal of the ligated tissue where adequate granulation is still not available would lead to muscularis sloughing and may be a risk for subserosal tissue exposure.

In the comparison of the two devices, although endoscopic visualization was apparently better with the J‐EBL (11.8 mm) than with the W‐EBL (8.6 mm), which may reduce the stress of endoscopists during EBL procedures, the insertion time to the deepest colonic portion was not statistically different between the two devices. Notably, the number of attempts to insert the device into the deepest colonic portion in the present study was small; thus, further study is necessary by increasing the number of cases. Statistical differences were not observed in the macroscopic and histological outcomes between the J‐EBL and the W‐EBL. Regarding the size, because the size of colonic diverticula varies, it is assumed that more diverticula may be accommodated by the J‐EBL because of its larger inner hood diameter. However, continuous banding cannot be performed with the J‐EBL, and reinsertion is necessary when banding is missed. In contrast, the W‐EBL has a limited endoscopic view but can be used continuously. This is an advantage of the W‐EBL when banding is missed. In this experiment, the total procedure time was significantly shorter with the W‐EBL. This is considered to be a result of the experimental design. Namely, we planned to perform EBL sequentially, and the number of scope reinsertions was low with the W‐EBL because it can perform up to seven ligations continuously. It is desirable that the EBL device is selected according to the patients' condition. For example, the J‐EBL can be used both for left‐ and right‐sided colonic bleeding because of its wide endoscopic view, whereas in cases of sigmoid colonic bleeding, W‐EBL may be appropriate because of the possibility for continuous use when banding is missed.

EBL is a widely used treatment method in humans. In our institution, more than 250 procedures have been performed. Prior retrospective cohort studies have reported a 96% success rate, 9–12% early rebleeding rate,^7^, ^15^ and no perforation. The results of this animal study corroborate the safety of both the J‐EBL and the W‐EBL.

The present study had several limitations. First, it was a single‐center study with a relatively small sample size. Second, we used an in vivo animal model without diverticula, meaning that EBL was performed on a normal colon, and not on sites that lack the muscularis propria layer. Third, the observation period may not have been sufficient to observe the complete healing of the colonic mucosa after EBL. Despite these limitations, we strongly believe that the findings of this study could be applied to humans. Because the normal colonic mucosa around the diverticulum is ligated by the band, evaluation of the banded layer is important, regardless of the diverticulum. In this study, ligation of the muscularis propria layer did not result in perforation at days 1 or 7. We suggest that the results of this animal study could relate to CDB. Nonetheless, further human studies are needed to demonstrate the effectiveness and safety of the two devices for CDB treatment.

In conclusion, both evaluated devices were safe for EBL. Macroscopic and pathological differences were not observed between the two devices or between days 1 and 7. Ligation of the muscularis propria layer did not result in perforation. The suitable device can be selected according to the patients' clinical condition.
